# Absence of Bacteria Permits Fungal Gut-To-Brain Translocation and Invasion in Germfree Mice but Ageing Alone Does Not Drive Pathobiont Expansion in Conventionally Raised Mice

**DOI:** 10.3389/fnagi.2022.828429

**Published:** 2022-07-18

**Authors:** Aimée Parker, Steve A. James, Catherine Purse, Arlaine Brion, Andrew Goldson, Andrea Telatin, David Baker, Simon R. Carding

**Affiliations:** ^1^Gut Microbes and Health Research Programme, Quadram Institute, Norwich, United Kingdom; ^2^Norwich Medical School, University of East Anglia, Norwich, United Kingdom

**Keywords:** *Candida albicans*, gut-brain, ITS1 sequencing, mycobiome, pathobiont, dementia, ageing

## Abstract

Age-associated changes in the structure of the intestinal microbiome and in its interaction with the brain via the gut-brain axis are increasingly being implicated in neurological and neurodegenerative diseases. Intestinal microbial dysbiosis and translocation of microbes and microbial products including fungal species into the brain have been implicated in the development of dementias such as Alzheimer’s disease. Using germ-free mice, we investigated if the fungal gut commensal, *Candida albicans*, an opportunistic pathogen in humans, can traverse the gastrointestinal barrier and disseminate to brain tissue and whether ageing impacts on the gut mycobiome as a pre-disposing factor in fungal brain infection. *C. albicans* was detected in different regions of the brain of colonised germ-free mice in both yeast and hyphal cell forms, often in close association with activated (Iba-1^+^) microglial cells. Using high-throughput ITS1 amplicon sequencing to characterise the faecal gut fungal composition of aged and young SPF mice, we identified several putative gut commensal fungal species with pathobiont potential although their abundance was not significantly different between young and aged mice. Collectively, these results suggest that although some fungal species can travel from the gut to brain where they can induce an inflammatory response, ageing alone is not correlated with significant changes in gut mycobiota composition which could predispose to these events. These results are consistent with a scenario in which significant disruptions to the gut microbiota or intestinal barrier, beyond those which occur with natural ageing, are required to allow fungal escape and brain infection.

## Introduction

Ageing is the dominant risk factor associated with the development of neurodegenerative dementias. Altered intestinal microbiota structure and function (microbial dysbiosis) with age is considered a contributing factor in the development of age-associated chronic low-grade systemic and tissue inflammation, termed inflammageing (Boulangé et al., 2016; Fransen et al., 2017; Thevaranjan et al., 2017; Boehme et al., 2021; Parker et al., 2021) which contributes to neuroinflammation and neurodegenerative disease (Scott et al., 2017; Boehme et al., 2019). Whilst bacterial community diversity, composition, and function changes significantly with age in both animal models and in humans (Claesson et al., 2011; Yatsunenko et al., 2012; Langille et al., 2014; Clark et al., 2015; O’Toole and Jeffery, 2015), comparatively little is known about the impact of ageing upon other members of the intestinal microbiota, including viruses, archaea, and fungi. Fungal diversity in the gut microbiome is decreased in adults compared to infants and children, with fungal richness being higher in females than males regardless of age (Strati et al., 2016). However, little is known of the intestinal fungal composition of elderly versus young adults, or whether fungal composition is altered due to ageing *per se*, or results from changes in behaviour and lifestyle, which occur concomitantly with ageing.

Fungi account for a relatively small fraction of the total human faecal microbiota (10^5^–10^6^ cells/g faecal matter compared with 10^11^ bacterial cells/g) (Huseyin et al., 2017) and for around 0.1% of the faecal microbiota gene content (Qin et al., 2010; Li et al., 2014; Sender et al., 2016). However, this is likely to be an underestimate of the true fungal intestinal load due to the comparatively smaller number of fungal reference genomes currently available, bias in microbiome analyses introduced by extraction and sequencing methods sub-optimal for mycobiome characterisation (Richard and Sokol, 2019), and the issue that faecal sampling is unlikely to accurately reflect fungal load throughout the GI tract and at the epithelial surface.

Fungal pathogens acquired externally to the host, and reactivation of latent infections, can lead to systemic fungal infection, resulting in significant pathology and mortality (Brown et al., 2012). In some circumstances, fungal species within the gut microbiota, which are normally well tolerated, may disseminate via the circulation to other sites including the brain. For example, cryptococcal meningoencephalitis can occur in immunocompromised individuals or those undergoing specific drug treatment, as well as in premature infants of very low birth weight (Gottfredsson and Perfect, 2000). Invasive candidiasis is a potentially life-threatening fungal infection caused by several *Candida* species, the most common being *Candida albicans*, a dimorphic fungus, which is a common human gut commensal (Brown et al., 2012). When able to penetrate the body’s barrier sites, *C. albicans* can cause superficial mucosal infections, and in some cases severe systemic sepsis with associated mortality exceeding 70% (Brown et al., 2012; Allert et al., 2018).

Increased risk of developing Alzheimer’s disease (AD) has been associated with infections of the central nervous system (CNS), potentially via impacting innate immune mechanisms and/or protein misfolding (Mawanda and Wallace, 2013). Viral, bacterial, and fungal species have been investigated in this context (Hammond et al., 2010; Huang et al., 2014; Fung et al., 2017; Dominy et al., 2019; Tetz et al., 2020); however, no single infectious agent has to date been demonstrated to be causative in AD onset. Fungal antigens from a variety of species have been detected in the serum of AD patients, including *C. albicans* and a number of other *Candida* species (Pisa et al., 2015b; Alonso et al., 2018). In addition, analysis of post-mortem brain tissue from AD patients and healthy controls identified genetic material from multiple fungal species (including *Candida*), fungal proteins, and fungal cell bodies unique to the brains of AD patients (Alonso et al., 2014; Pisa et al., 2015a,b).

Despite these findings, the concept of a brain-associated microbiota remains highly controversial, and there is no compelling evidence of microbial representation in the CNS of normal healthy hosts. More plausible is that microbes, including fungi, escape confinement in the gut or elsewhere and disseminate more widely when barrier sites and/or the immune system have been seriously compromised. In ageing, declining immune function (immunosenescence), inflammageing, intestinal microbial dysbiosis, and the high incidence of co-morbidities create an environment more permissive to microbial translocation to the circulatory system and dissemination to tissues beyond the gastrointestinal tract (GIT).

Animal studies of the mycobiome and fungal infection can control for or eliminate most of the confounding factors which complicate interpretation of fungal changes in ageing human populations. Mice, for example, harbour many of the same fungal taxa which inhabit the human gut, with a characteristic feature of both the murine and human gut mycobiome being the dominance of the Ascomycota and Basidiomycota phyla (Hallen-Adams et al., 2015; Nash et al., 2017; Ward et al., 2018; Doron et al., 2019; James et al., 2020; Mims et al., 2021). *C. albicans* is frequently present in the healthy human gut as a benign commensal (Brown et al., 2012; Nash et al., 2017; James et al., 2020) and can also be found in captive-bred mice (Doron et al., 2019; Mims et al., 2021), although it may be absent in wild murine species (Bendová et al., 2020).

Multiple *Candida* species can colonise mouse models and persist in the GIT (Prieto and Pla, 2015). Systemic dissemination and candidiasis is evident in immunocompromised mice, however, this often requires high initial inoculums for non-*albicans Candida* species (Conti et al., 2014; Segal and Frenkel, 2018). *C. albicans* can also stably colonise mice and has been used to study fungal intestinal colonisation and dissemination in neonatal mice, antibiotic- or chemotherapy-treated adult mice, and germ-free mice (Field et al., 1981; Kinneberg et al., 1999; Mellado et al., 2000; Wiesner et al., 2001; Schofield et al., 2005; Koh et al., 2008; Koh, 2013). When administered intravenously, *C*. *albicans* can infect the mouse brain and cause localised cerebritis (Wu et al., 2019).

**FIGURE 1:**
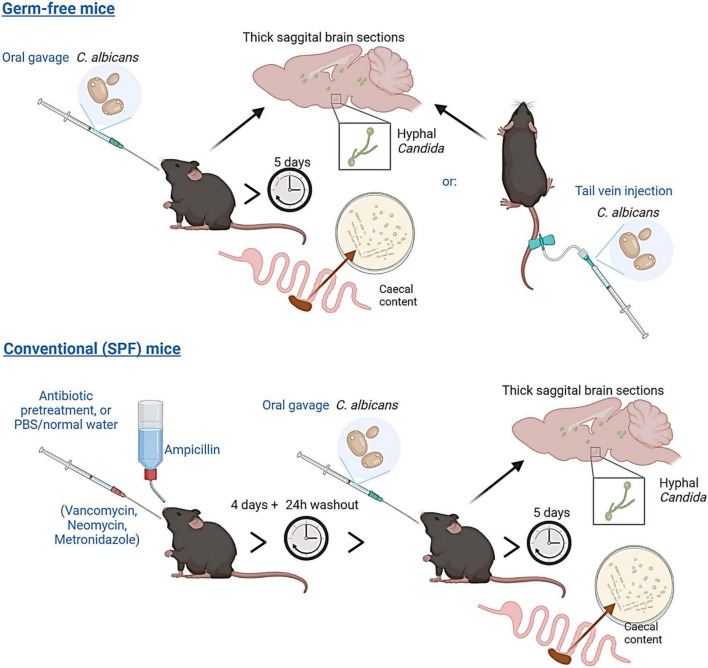
Experimental overview. Germ-free C57BL/6 mice were administered *Candida albicans* (strain NYCY 3115) either by oral gavage or tail vein injection. Conventional (Specific-Pathogen-Free, SPF) mice were pre-treated with a broad-spectrum antibiotic cocktail (or delivered PBS/normal drinking water) for 5 days, prior to delivery of *Candida albicans* by oral gavage. Caecal content and brain tissues from all mice were collected five days post *C. albicans* delivery to assess colonisation and dissemination to the brain.

Here we assessed whether fungal cells could traverse the intestinal barrier and disseminate to the brain by colonising C57BL/6 germ-free mice with a human-derived isolate of *C. albicans* by oral gavage, using confocal microscopy to assess fungal cell dissemination throughout the brain (a graphical overview is shown in Figure 1). High-throughput amplicon sequencing of the fungal internal transcribed spacer 1 (ITS1) region was used to investigate the effect of ageing on the composition and diversity of the murine gut mycobiome, and to identify potential fungal pathobionts.

## Materials and Methods

### Yeast Strain and Growth Conditions

*Candida albicans* strain (NCYC 3115) is a human clinical isolate from patient faeces collected in a United Kingdom hospital and was provided by the National Collection of Yeast Cultures (Norwich, United Kingdom). For inoculum preparation, stocks were cultured in YM liquid medium (10g/L glucose, 3g/L malt extract, 5g/L peptone, 3g/L yeast extract) at 30°C for 48h with shaking (200 rpm). Cells were collected by low-speed centrifugation (3,000 rpm, 5 min), washed twice in sterile phosphate buffered saline (PBS) and re-suspended in PBS prior to delivery to mice. Fungal colonisation was assessed by measuring CFUs (colony forming units) of *C. albicans* present in the caecum of each mouse. Caecal contents, collected five days post-delivery, were mechanically homogenised in PBS to 100 mg/mL then serially diluted and spread plated onto YM medium. All agar plates were incubated aerobically at 37°C, and colony counts measured after 2 days incubation. Colony morphology was also assessed (and counts determined) by visual inspection, with colonies of differing morphology (morphotypes) selected and stocked for additional phenotyping. YM broth cultures derived from two, post-passaged, colony morphotypes (white and domed vs. darker and flattened) were incubated at 37°C, without agitation, and examined after 3 days by standard light microscopy for the presence/absence of hyphal and pseudohyphal cells. Species identity was confirmed by standard colony PCR using *C. albicans*-specific primers (Asadzadeh et al., 2018), and by ITS1 sequencing (White et al., 1990; Gardes and Bruns, 1993). Details of all fungal primers used in this study are provided in Supplementary Figure 4.

### Animal Experiments

All experiments involving animals were performed in accordance with EU and United Kingdom Home Office Legislation and local Animal Welfare and Ethical Review Body approval. Male and female specific pathogen-free (C57BL/6 -SPF) mice aged 3 months or 24 months, and male germ-free (C57BL/6-GF) mice aged 3 months, were maintained in individually ventilated cages (SPF) or in sterile isolators (GF) in adjacent rooms of the Quadram Institute Germ-Free mouse facility within the University of East Anglia Disease Modelling Unit. All mice received autoclaved water and were fed RM3 (SPF) or RM3-(Autoclavable) (GF) diet (Special Diets Services). All mice were maintained under 12-h light-dark cycle. A dose of 2.5 × 10^5^ (*n* = 5) or 5 × 10^5^ (*n* = 5) *C. albicans* cells re-suspended in 200 μL PBS was administered to germ-free animals by oral gavage, whilst a lower dose of 2.5 × 10^4^ cells in 100 μL of PBS was used for tail vein injection control mice. SPF mice (*n* = 16, 8 females and 8 males) were pre-treated for four days with either PBS (*n* = 8) or a cocktail of broad-spectrum antibiotics (VMNA, 0.5 mg/mL vancomycin, 1 mg/mL metronidazole and 1 mg/mL neomycin delivered in 200 μL sterile water by daily oral gavage, and 1 mg/mL ampicillin delivered via drinking water, available *ad libitum*), *n* = 8. Following a 24 h washout period 5 × 10^5^ *C. albicans* cells re-suspended in 200 μL PBS were administered by oral gavage. Mice were then maintained in individually ventilated cages until sacrifice. Brains and caecal content were harvested at day 5 post-inoculation and used for downstream analysis.

Formalin-fixed paraffin-embedded brains were sectioned at 5 μm. Sagittal vibratome sections of 100 μm thickness were prepared from PFA-fixed whole brains embedded in low-melt agarose, a method adapted from Snippert et al. (2011) and were cleared post-staining and prior to mounting using RapiClear (CamBioscience, Cambridge, United Kingdom). *C. albicans* was visualised in sections using a rabbit polyclonal anti-*C. albicans* antibody (NB100-64750 Novus Biologicals, 1:100), and for activated microglia/macrophages using rabbit anti-Iba-1 (ab178846, Abcam, 1:100) for single staining or Abcam ab150167 (1:100) for co-stains. Secondary antibodies used were goat anti-rabbit IgG Alexa Fluor-594 (Invitrogen, 1:100), Goat Anti-Rat Alexa-647 (ab150167, Abcam, 1:500) or donkey anti-rabbit IgG Alexa Fluor 488 (Invitrogen, 1:500). Nuclei were stained with Hoechst 33258. Images were collected and analysed using a Zeiss LSM880 confocal microscope and ZEN 2010 software, and FIJI/ImageJ v2.1.0 (Schindelin et al., 2012). *C. albicans* cells were quantified from 100 μm sagittal vibratome sections taken starting from the midline of the left hemisphere of the brain, five sections were taken from each brain sample from *C. albicans*-colonised germ-free mice (*n* = 5 mice), non-colonised control germ-free mice (*n* = 3 mice) and from *C. albicans*-colonised SPF mice receiving either antibiotic or PBS only pre-treatment (*n* = 8 mice/group). Cells were not included in counts if they were obviously within vessels, or were on the periphery of the section and therefore considered to not be truly within the brain tissue.

### Genomic DNA Extraction

Faecal pellets were collected from temporarily singly housed SPF mice using sterile picks and sterile RNA-DNA-free microtubes and were stored at –70°C prior to processing and DNA extraction. For fungal DNA amplification, total microbial DNA was extracted from ∼50 mg of faeces from each animal using the QIAamp PowerFecal Pro DNA kit (QIAGEN, Hilden, Germany) and following the manufacturer’s protocol. In addition, all samples were homogenised using a FastPrep-24 benchtop instrument (MP Biomedicals, Irvine, CA, United States) at 6.0 m/s for 1 min. Extracted DNA was quantified and quality checked using the Qubit 3.0 fluorometer and associated Qubit dsDNA BR Assay Kit (Thermo Fisher, Waltham, MA, United States). DNA samples were stored at –20°C prior to further analysis.

### Internal Transcribed Spacer 1 Amplification and Sequencing

The fungal ITS1 region was amplified from 100 ng of faecal DNA by using the pan-fungal ITS1F and ITS2 primer set (White et al., 1990; Gardes and Bruns, 1993), with each primer modified at the 5’ end to include an Illumina adapter tail, using KAPA2G Robust DNA polymerase (Kapa Biosystems, Wilmington, MA, United States). Amplification was performed at 94°C (5 min) with 35 cycles of 92°C (30 s), 55°C (30 s), 72°C (45 s), and a final extension of 72°C (5 min). Amplification reactions were set up in duplicate for each DNA sample, and negative (PCR dH_2_O) and positive controls (0.01 ng of *C. albicans* DNA) were included in each PCR run. Following ITS1 PCR, a 0.7x SPRI purification using KAPA Pure Beads (Roche, Wilmington, MA, United States) was performed and the purified DNA was eluted in 20 μl of 10 mM Tris-HCl. In a second PCR, library index primers were added using a Nextera XT Index Kit v2 (Illumina, Cambridge, United Kingdom) and amplification was performed at 95°C (5 min) with 10 cycles of 95°C (30 s), 55°C (30 s), 72°C (30 s), and a final extension of 72°C (5 min). Following PCR, libraries were quantified using the Invitrogen™ Quant-iT dsDNA high sensitivity assay kit (Thermo Fisher) and run on a FLUOstar Optima plate reader (BMG Labtech, Aylesbury, United Kingdom). Libraries were pooled following quantification in equal quantities. The final pool was SPRI cleaned using 0.7x KAPA Pure Beads, quantified on a Qubit 3.0 fluorometer and run on a High Sensitivity D1000 ScreenTape (Agilent Inc., Santa Clara, CA, United States) using the Agilent Tapestation 4200 to calculate the final library pool molarity. The pool was run at a final concentration of 8 pM on an Illumina MiSeq instrument using the MiSeq^©^ v3 (2 × 300 bp) Kit (Illumina). All sequencing was performed at Quadram Institute Bioscience, Norwich. The raw data were analysed locally on the MiSeq instrument using MiSeq reporter.

### Mycobiome Characterisation

Illumina MiSeq reads were analysed using the automated pipeline Dadaist2, a dedicated workflow for ITS profiling (Ansorge et al., 2021). The quality profile of the raw reads (in FASTQ format) was assessed using *Fastp* 0.20.0 (Chen et al., 2018), which was also used to remove reads with ambiguous bases. Locus-specific primers were removed using *SeqFu* 1.8 (Telatin et al., 2021). The identification of representative sequences was performed using DADA2 (Callahan et al., 2016), to produce a set of amplicon sequence variants (ASVs), and their taxonomic assignment was determined using the UNITE Fungal ITS database (release 8.2) (Nilsson et al., 2019). The multiple alignment of the representative sequences was performed using ClustalO (Sievers and Higgins, 2021) and the guide tree was produced using FastTree (Price et al., 2009). Data normalization and diversity were produced using the Rhea scripts (Lagkouvardos et al., 2017). The output feature table, taxonomic classification, phylogeny and metadata files were exported and further analysed using MicrobiomeAnalyst (Dhariwal et al., 2017) and the built-in plotting provided by Dadaist2. Every ASV with a zero count in all samples was removed to assess alpha diversity measures.

### Statistical Analysis

Three alpha-diversity measures were used to estimate fungal taxa richness (Chao1) as well as taxa richness and evenness (Shannon and Simpson) using MicrobiomeAnalyst (Dhariwal et al., 2017). Data was not rarefied, was scaled by total sum scaling, was non-transformed, and statistical significance was assessed by Student’s *t*-test (threshold for significance *P* < 0.05). For comparison of specific taxa, data were CLR-transformed prior to comparison between two groups by *t*-test.

### Other Software

Figure 1 was created using BioRender illustration software: https://biorender.com/.

## Results

### *Candida albicans* Translocates From Gut to Brain in Monocolonised Germ-Free Mice and Induces an Inflammatory Response in the Brain

**FIGURE 2:**
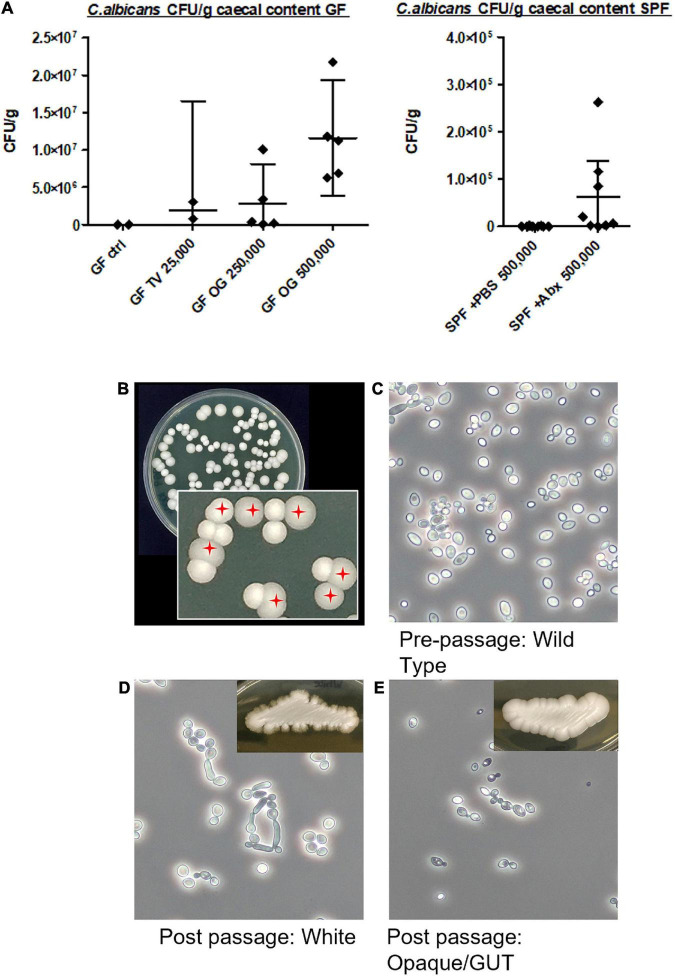
Colonisation of the caecum by *Candida albicans* NCYC 3115 following tail vein injection or oral gavage. **(A)** Colony-forming units (CFU) recovered from caecal content following delivery of *C. albicans* NCYC 3115 to germ-free mice (left) or SPF mice (right). No colonies were present in the caecal content of germ-free control mice administered PBS alone (GF ctrl). TV, tail vein; OG, oral gavage. Numbers on x-axis labels denote amount of *C. albicans* cells administered, error bars denote 95% CI. **(B)** Example YM agar plate with zoom inset showing two phenotypically distinct *C. albicans* colony morphotypes recovered from caecal contents. White and domed morphotype and darker and flattened/Gastrointestinally indUced Transition (GUT) morphotype (red crosshairs). **(C–E)** Photomicrographs of pre-passage wild type cells **(C)**, post-passage white phenotype cells **(D)**, and post-passage darker/GUT phenotype cells **(E)**, all grown at 37°C for 3 days in YM broth.

*Candida albicans* (NCYC 3115) was administered by oral gavage to two groups of germ-free adult C57BL/6 mice, in doses of either 2.5 × 10^5^ or 5 × 10^5^ cells. A third group were administered an inoculum of 2.5 × 10^4^ cells by tail vein injection, a dose previously shown to result in fungal translocation to the brain with no lethality (Wu et al., 2019). Control mice received PBS alone by gavage. Both delivery routes, oral or intravenous, resulted in successful colonisation of the GIT, as measured by CFU recovered from caecal content five days post-delivery (Figure 2A). Oral administration of 2.5 × 10^5^ cells resulted in caecal counts ranging from 1 × 10^5^ to 1 × 10^7^CFU, whereas administration of the higher dose of 5 × 10^5^ cells resulted in caecal counts ranging from 6.2 × 10^6^ to 2.2 × 10^7^CFU. Mice receiving yeast cells intravenously had lower caecal CFU counts of 8 × 10^5^ – 3 × 10^6^. Caecal content from control mice receiving PBS alone yielded no fungal colonies. Species identity of colonies was confirmed by standard colony PCR using *C. albicans*-specific primers (Asadzadeh et al., 2018).

Two types of post-passage *C. albicans* colonies were cultured from the caecal contents (Figures 2B–E), a white and domed morphotype (as per the wild-type), and a darker and flattened morphotype, chromatically and morphologically resembling the previously described Gastrointestinally indUced Transition (GUT) phenotype (Pande et al., 2013). Approximately 66% of colonies recovered from the caecal content of mice in the present study were of this ‘GUT’-like phenotype, suggesting substantial adaptation of the administered wild-type *C. albicans* to the C57BL/6 germ-free gut. Cultures derived from this phenotype failed to produce hyphae, either on solid or in liquid media, when grown at 37°C. This was in marked contrast to white phenotype-derived cultures which readily produced hyphae (and pseudohyphae) when grown at this elevated temperature (data not shown).

**FIGURE 3:**
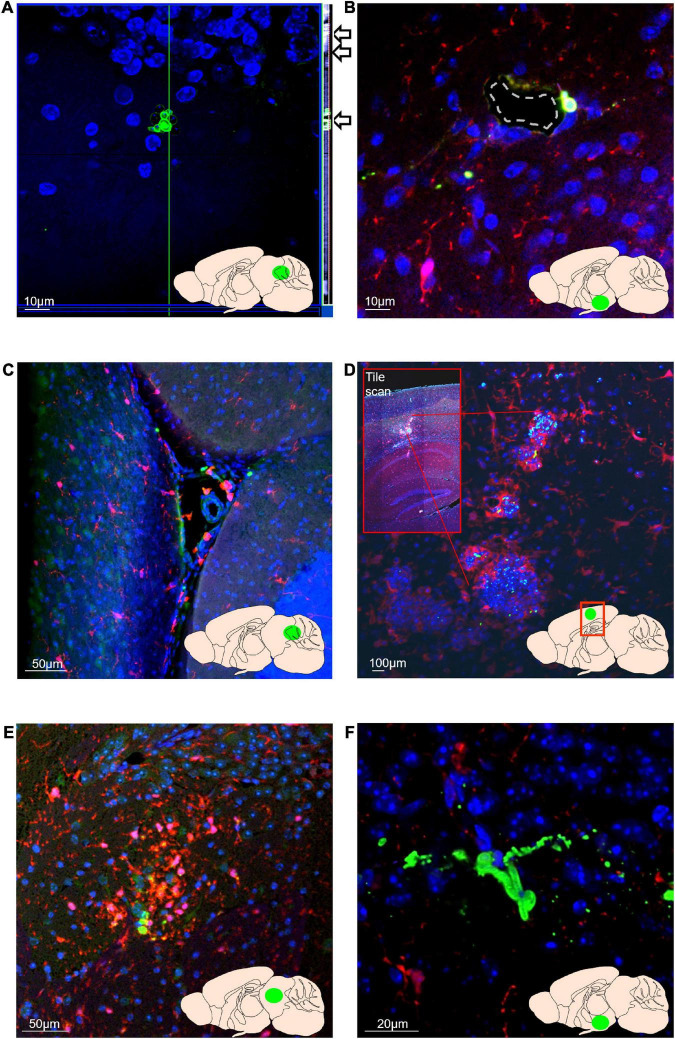
*Candida albicans* can disseminate from the gut to the brain and can grow in the invasive hyphal form within brain tissue. In all images, blue = nuclei (Hoechst), green = *C. albicans*, red = Iba-1-positive microglia/macrophages. Green area in inset brain schematic indicates approximate position of image. **(A)** *C. albicans* cells are detectable within cerebellar brain tissue at 5 days post-colonisation. Z-stack orthogonal view (side bar and arrows) shows *C. albicans* cells are in the same plane as brain cell nuclei. Orthogonal side bar brightness and contrast has been enhanced here for visibility. **(B)** *C. albicans* cells in proximity to a hypothalamic blood vessel (dashed outline). **(C)** *C. albicans* cells and Iba-1 positive macrophages within the cerebral aqueduct (lobule II granule layer visible as dense Hoechst-stained area bottom right of image). **(D)** Foci of clustered Iba-1^+^ cells (red) around *C. albicans* cells (green) within the posterior parietal association area of the cortex. Inset box shows overview tile scan of the cortex and hippocampus. **(E)** Cluster of Iba-1 + cells around *C. albicans* cells within the midbrain **(F)** Entwined hyphal *C. albicans* hyphae within the hypothalamus.

In mice receiving *C. albicans* orally of either lower (2.5 × 10^5^) or higher dose (5 × 10^5^) inoculum, and in mice receiving the inoculum intravenously, *C. albicans* cells were detected in brain tissue five days post-colonisation by immunostaining with an anti-*C. albicans* antibody (Figures 3A–F). Individual *C. albicans* cells and cell clusters were found throughout the brain, in the ventricular spaces, cerebellum, hypothalamus, midbrain and cortex. Clusters and individual *C. albicans* cells were confirmed to be within the plane of the brain tissue by imaging of z-stacks (Figure 3A). Individual *C. albicans* cells and cell clusters were frequently found in, or adjacent to, vessels within the brain tissue (Figure 3B), and within the ventricular spaces, including the cerebral aqueduct (Figure 3C). *Candida albicans* cells were frequently surrounded by Iba-1^+^ cells resembling both resident microglia and infiltrating macrophages within or exiting vessels (Figures 3D,E), indicating induction of an inflammatory microglial/macrophage response. In one mouse, striking granuloma-like clusters of fungal and Iba-1^+^cells were seen in the posterior parietal cortex (Figure 3D), which plays a key role in spatial representation of objects for action planning and control. Less frequently hyphae were detected within brain tissue samples (Figure 3F) indicating *C. albicans* cells were viable and in an invasive form. No fungal cells or similar microglial clusters were observed in PBS control germ-free mouse brain samples. As mice were not transcardially perfused before brain harvest, we cannot completely rule out that a small number of counted *C. albicans* cells may have been within vessels/capillaries that were sectioned or optically sliced in such a way that we did not identify the vessels. However, the identification of hyphal forms within the brain tissue, and clusters of microglia identified surrounding *C. albicans* cells strongly suggests active invasion of the brain tissue as opposed to circulating yeast cells in dissemination form (Gow et al., 2011; Noble et al., 2016).

### Short-Term Depletion of Gut Bacteria in Conventional Mice Permits Expansion of *Candida albicans* in the Caecum

To test whether depletion of the gut bacterial community in conventional mice would also allow for fungal expansion and dissemination, we pre-treated SPF mice with a short course of broad-spectrum antibiotics (VMNA), or PBS, prior to *C. albicans* delivery by oral gavage (Figure 1). Colony counts from caecal content (Figure 2A) showed increased caecal colonisation in antibiotic-pre-treated SPF mice (SPF + Abx) compared with PBS-pre-treated SPF controls, but at much lower levels compared to *C. albicans*-colonised germ-free mice (mean 1.15 × 10^7^ cells/g caecal content in colonised germ-free versus 6.15 × 10^4^ in colonised SPF + Abx). On analysing the brains of the SPF mice, we found no evidence of fungal cells, either in yeast or hyphal form, within brain sections of either PBS control or antibiotic pre-treated colonised mice, either by staining specifically for *Candida*, or by using a non-specific fungal cell wall stain (example expected staining of positive control shown in Supplementary Figure 1B).

Our data shows that disruption of the intestinal environment by antibiotic treatment permits increased fungal colonisation of the intestinal tract, but suggest that short term-antibiotic treatment is not sufficient to promote dissemination to the brain. On the other hand, recent data show that long-term chronic administration of antibiotics can promote systemic dissemination of both fungi and bacteria (Drummond et al., 2022). Advanced age is also associated with changing gut bacterial composition, as well as depleted barrier integrity promoting chronic systemic inflammageing (Fransen et al., 2017; Thevaranjan et al., 2017; Parker et al., 2022). Therefore, we next investigated whether the composition of the enteric mycobiota is altered in aged animals, and whether any fungal species detected are potential pathobionts/opportunistic pathogens with the capacity to cause serious infection.

### Ageing Alone Is Not Sufficient to Select for or Drive Pathobiont Expansion in Specific Pathogen-Free Mice

High-throughput internal transcribed spacer 1 (ITS1) amplicon sequencing was used to characterise the faecal fungal communities in young (3-month) and aged (24-month) SPF mice. A total of 1,471,212 quality-trimmed ITS1 reads were obtained, ranging from 14,913 (A7, aged cohort) to 100,384 (Y2, young cohort), with a sample average of 73,560 reads (Supplementary Figure 2A). Over 2,000 amplicon sequence variants (ASVs) were used to determine the composition of the fungal microbiota in the young and aged mice at different taxonomic levels.

**FIGURE 4:**
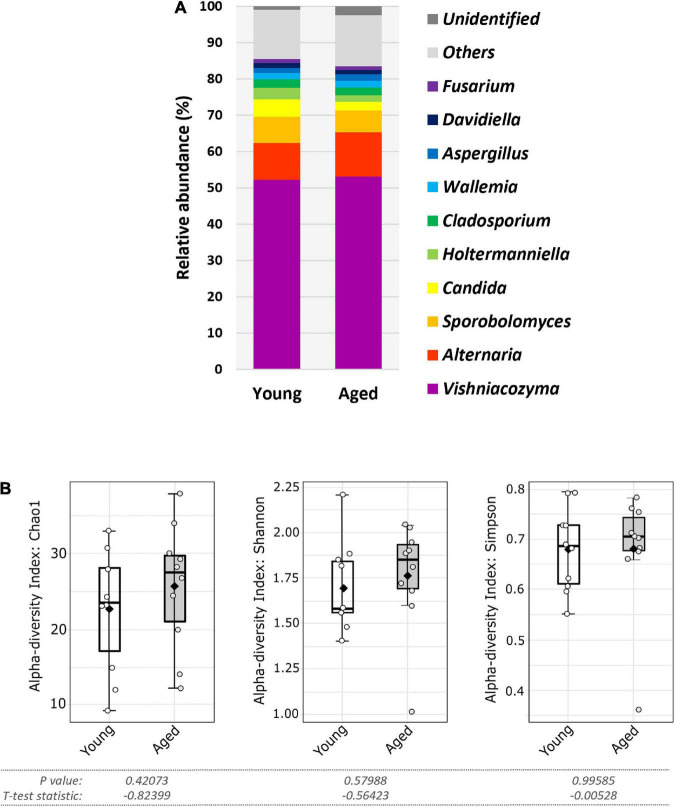
Faecal fungal diversity and top ten genera in aged vs. young SPF mice. **(A)** Top ten most abundant genera (percentage mean relative abundance) in faecal samples of young vs. aged SPF mice (*n* = 10/group). **(B)** Alpha diversity (L-R: Chao1, Shannon, and Simpson indices) of faecal fungal composition of young vs. aged SPF mice (*n* = 10/group), whiskers show spread of data across all mice, solid black dot indicates the mean, horizontal line indicates the median.

At the phylum level, most identified fungi in each age group belonged to either the Ascomycota or Basidiomycota (Supplementary Figure 2B). A characteristic feature of the gut mycobiome of our C57BL/6 colony, irrespective of age, was the predominance of the Basidiomycota. At the genus level, when analyses were restricted to the most abundant genera (i.e., those with a relative abundance of 1% or more), which accounted for over 80% of all ITS1 reads, both age groups had broadly similar taxonomic profiles, with *Vishniacozyma* the predominant genus (Figure 4A). This basidiomycetous yeast genus had a mean relative abundancy of over 50% in each age group (young, 52.1%; aged, 53.1%). Other notable genera included *Alternaria*, *Sporobolomyces*, *Candida*, *Holtermanniella*, and *Cladosporium* (Figure 4A). Whilst most genera displayed comparable mean relative abundancies in both age groups (Supplementary Figure 2B), *Sporobolomyces*, *Candida* and *Holtermanniella* were all nominally less abundant, albeit not reaching statistical significance, in the aged mice (Figure 4A and Supplementary Figure 2B). At the genus level, there was no significant compositional change in alpha diversity between the two age groups (*p* > 0.05 in all three indices) (Figure 4B).

**FIGURE 5:**
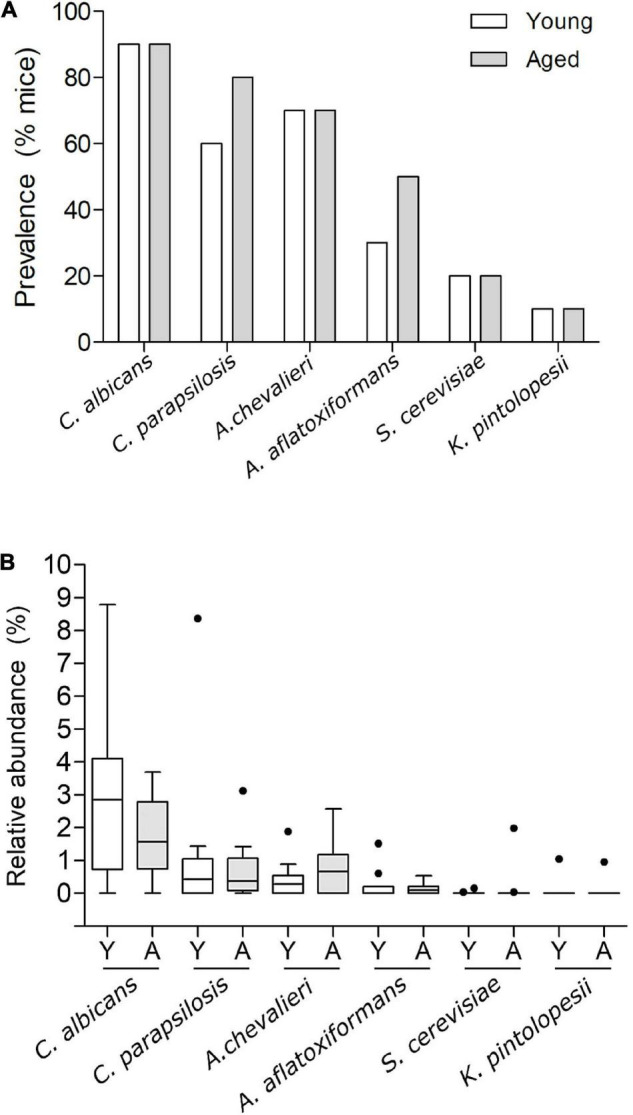
Prevalence and relative abundance of putative gut commensal fungal species in faecal samples of young vs. aged SPF mice. **(A)** Prevalence of putative gut commensal fungal species *Candida albicans*, *Candida parapsilosis, Aspergillus chevalieri*, *Aspergillus aflatoxiformans*, *Saccharomyces cerevisiae*, and *Kazachstania pintolopesii* in faecal samples from groups of young and aged SPF mice (*n* = 10/group), percentage of mice harbouring each species displayed as % prevalence. **(B)** Percentage relative abundance of those same species in faecal samples from aged (A) vs. young (Y) mice (*n* = 10/group), Tukey whiskers, horizontal bars show the mean, outliers displayed as round points.

For taxa resolved to species level most were categorised as environmental fungi, typically found in soil and/or plant associated. This included *Vishniacozyma victoriae*, the most abundant taxon and a species present in every sample (Supplementary Figure 3). Six species were identified as candidate gut colonisers based on their ability to survive and proliferate at 37°C. These were *Aspergillus aflatoxiformans*, *Aspergillus chevalieri*, *Candida albicans*, *Candida parapsilosis*, *Kazachstania pintolopesii*, and *Saccharomyces cerevisiae*. Among these, *C. albicans* was the most prevalent in both age groups (Figure 5A), albeit at lower nominal relative abundance in the elderly mice compared to young mice (Figure 5B and Supplementary Figure 3), (young, 2.9%; aged 1.7%), although this was not statistically significant (*p* > 0.05) (Figure 5B, and Supplementary Figure 3). In contrast, *A. chevalieri*, which displayed similar prevalence in both cohorts (70%), was present at nominally higher relative abundance in the aged mice (young, 0.42%; aged, 0.77%), although this was also not statistically significant (*p* > 0.05) (Figure 5B and Supplementary Figure 3). *K. pintolopesii* a common rodent-associated yeast species (Kurtzman et al., 2005; Bendová et al., 2020) was found in only two of the mice (one from the 3-month-old group and one from the 24-month-old group), and at relatively low abundance (∼1%).

In summary, although the overall enteric mycobiota profiles of young and aged mice were broadly similar at the genus level, subtle differences in both the prevalence and abundance were evident at the species level. These differences were evident within a small group of putative commensal fungi, which included three opportunistic pathogens.

## Discussion

There is growing interest in the concept that intestinal microbial dysbiosis, as well as microbial infection, contributes to neuroinflammation and neurodegenerative disease, including dementias (Fung et al., 2017; Vogt et al., 2017). The majority of such studies have focused almost exclusively on the prokaryome, with only a small number investigating the mycobiome and implicating fungi in neurological disorders and AD (Alonso et al., 2014; Pisa et al., 2015a,b; Fung et al., 2017; Forbes et al., 2018; Ling et al., 2020). Gut-resident *C. albicans* populations represent the principal source of life-threatening disseminated candidiasis (Bougnoux et al., 2006; Gouba and Drancourt, 2015). In the gut, pathological invasion of *C. albicans* across the epithelial barrier and into the bloodstream occurs via hyphal penetration of cells, hyphal production of a cytolytic peptide toxin (candidalysin), enterocyte necrosis and subsequent loss of epithelial barrier integrity (Dalle et al., 2010; Allert et al., 2018). Here, using oral delivery of *C. albicans* cells to the GIT of germ-free C57BL/6 mice, we demonstrate that *C. albicans* can traverse both the intestinal and blood-brain barriers and produce hyphae within the brain. We also observed clusters of Iba-1^+^ activated immune cells surrounding *C. albicans* cells, in accordance with prior reports of gliosis in mouse models of candidiasis (Lionakis et al., 2011; Wu et al., 2019). Hyphae were not found within the brains of SPF C57BL/6 mice administered *C. albicans* via intravenous injection (Wu et al., 2019), which may reflect the use of different isolates of *C. albicans* between studies, or differences in SPF vs. germ-free mice.

We also found that while short-term antibiotic pre-treatment allowed for increased expansion of *C. albicans* in colonised SPF mice, compared to PBS pre-treated controls, no fungal cells, either in yeast or hyphal form, were detected in the brains of the colonised mice. Drummond and colleagues (Drummond et al., 2022) have recently shown that chronic exposure to antibiotics (>4 weeks in mice or >7 days in humans) can promote fungal and bacterial dissemination to other organs, however, brains were not assessed for fungal cell staining in the mouse studies so it is unclear whether a longer antibiotic regimen might allow for dissemination into the brain tissues. SPF mice may be resistant to brain infection by *C. albicans*, as intestinal mucins can inhibit hyphal formation by *C. albicans* (Kavanaugh et al., 2014) and the mucus layer differs in composition between SPF and germ-free mice (Johansson et al., 2014; Jakobsson et al., 2015). Furthermore, differences in immune responses between *C. albicans* cells and macrophages (Erwig and Gow, 2016) in germ-free versus SPF mice may also affect hyphal formation and persistence.

Considering that fungal processes can contribute to intestinal barrier damage and that age-related intestinal dysbiosis may increase the likelihood of gut-to-brain translocation of microbes in older hosts, we compared the fungal mycobiome of young and aged mice. Within the mycobiota of young mice three species, namely *C. albicans*, *C. parapsilosis* and *K. pintolopesii*, are recognised opportunistic pathogens of humans and mice, and are capable of causing life-threatening systemic infections (Kurtzman et al., 2005; Pfaller and Diekema, 2007). However, the relative abundances of these species were not significantly different in aged mice, nor was there any evidence of significant fungal dysbiosis in aged mice. This suggests that ageing alone is not a major driver of fungal composition in the mouse gut microbiota. In mice at least, it is more likely that other environmental factors including dietary changes, medications (antibiotics), infections and/or changes in host defence mechanisms and immune status might be required to permit fungal gut-brain translocation in aged, but otherwise healthy, hosts.

There is limited available data on the mycobiota profile of aged healthy human adults (Strati et al., 2016), although some studies have sequenced the mycobiota of patients with metabolic or neurodegenerative disease (Ahmad et al., 2020; Jayasudha et al., 2020; Ling et al., 2020; Nagpal et al., 2020). The gut microbiome in patients with mild cognitive impairment (MCI) and those with AD is reported to differ from healthy controls (Vogt et al., 2017; Zhuang et al., 2018; Saji et al., 2019). A study of patients from a United States cohort with MCI for example, found a higher proportion of the fungal genera *Botrytis, Kazachstania*, *Phaeoacremonium*, and *Cladosporium* but a reduced proportion of *Meyerozyma* compared to controls (Nagpal et al., 2020). A study of the faecal mycobiome of a Chinese cohort of AD patients reported increased abundance of the species *C. tropicalis*, *Trametes versicolor*, *Schizophyllum commune*, *Davidiella tassiana*, *Exophiala dermatitidis*, and *Erythrobasidium hasegawianum* compared to controls, but found no significant differences in the most prevalent C*andida* species, including *C. albicans* (Ling et al., 2020). In both the MCI and AD cohorts, no significant change in fungal alpha or beta diversity was seen compared to controls (Ling et al., 2020; Nagpal et al., 2020). With no evident overlap between studies of shared taxa with altered relative abundance, it is currently not possible to identify specific fungi (e.g., pathobionts) which may be associated with the development of these neurodegenerative disorders.

A major difficulty in attributing causality in MCI or AD development to an altered microbiome or mycobiome is identifying and measuring confounding factors, in particular the impact of age-associated changes in lifestyle, diet, behaviour, and co-morbidities. For example, many prescribed orally administered drugs including antibiotics, antidepressants and anti-inflammatory compounds, can significantly impact microbiota composition and function (Maier et al., 2018, 2021; Vich Vila et al., 2020), as can behavioural changes and shifts in diet or living conditions (Auchtung et al., 2018; Raimondi et al., 2019). These factors are of relevance to patients with MCI or AD. Such co-variables are minimised in animal models kept under environmentally controlled conditions and maintained on defined diets. However, when using transgenic mouse models of AD for example, it is often unclear what effects the genetic modifications may have on host immune, neural, or other responses that create an altered intestinal environment which is permissive for particular microbes and pathobionts. Whilst these considerations may help explain conflicting results between human studies, and when comparing results of animal and patient studies, it remains to be determined whether altered microbiota and mycobiota composition is a contributing factor in the development of dementias, or is merely a symptomatic or correlative phenomenon.

## Conclusion

Here we show that in the absence of other enteric microbes, orally delivered *C. albicans* can translocate from the gut to the brain and induce cerebral inflammation. Furthermore, we also show that ageing alone did not alter the overall composition of the gut mycobiota in specific pathogen-free mice. This indicates that ageing alone is not sufficient to induce mycobiome dysbiosis and cerebral fungal infection, and that other disruptions to the gut microbiota and/or the intestinal barrier may be needed to permit gut fungal pathobiont escape and infection of the brain.

## Data Availability Statement

The datasets presented in this study can be found in online repositories. The names of the repository/repositories and accession number(s) can be found below: https://www.ncbi.nlm.nih.gov/, PRJEB49148.

## Ethics Statement

The animal study was reviewed and approved by local (University of East Anglia) Animal Welfare and Ethical Review Body approval. All experiments involving animals were performed in accordance with EU and United Kingdom Home Office Legislation, revised Animals (Scientific Procedures) Act 1986 United Kingdom.

## Author Contributions

AP and SJ: conceptualization, methodology, investigation, formal analysis, data visualization, manuscript original draft, and review and editing. CP: investigation, formal analysis, data visualization, and manuscript content and review. AB, AG, and DB: methodology and investigation. AT: methodology, investigation, and formal analysis. SC: supervision, resources, funding, project administration, and manuscript review and editing. All authors contributed to the article and approved the submitted version.

## Conflict of Interest

The authors declare that the research was conducted in the absence of any commercial or financial relationships that could be construed as a potential conflict of interest.

## Publisher’s Note

All claims expressed in this article are solely those of the authors and do not necessarily represent those of their affiliated organizations, or those of the publisher, the editors and the reviewers. Any product that may be evaluated in this article, or claim that may be made by its manufacturer, is not guaranteed or endorsed by the publisher.

## References

[B1] Ahmad H. F. Mejia J. L. C. Krych L. Khakimov B. Kot W. Bechshøft Rasmus L. (2020). Gut Mycobiome Dysbiosis Is Linked to Hypertriglyceridemia among Home Dwelling Elderly Danes. *BioRxiv*. [Epub ahead of print]. 10.1101/2020.04.16.044693

[B2] Allert S. Förster T. M. Svensson C. M. Richardson J. P. Pawlik T. Hebecker B. (2018). Candida Albicans-Induced Epithelial Damage Mediates Translocation through Intestinal Barriers. *MBio* 9:e915–e918. 10.1128/MBIO.00915-18/SUPPL_FILE/MBO003183909SF5.TIF PMC5989070 29871918

[B3] Alonso R. Pisa D. Fernández-Fernández A. M. Carrasco L. (2018). Infection of Fungi and Bacteria in Brain Tissue From Elderly Persons and Patients With Alzheimer’s Disease. *Front. Aging Neurosci.* 10:159. 10.3389/fnagi.2018.00159 29881346 PMC5976758

[B4] Alonso R. Pisa D. Marina A. I. Morato E. Rábano A. Carrasco L. (2014). Fungal Infection in Patients with Alzheimer’s Disease. *J. Alzheimer’s Dis.* 41 301–311. 10.3233/JAD-132681 24614898

[B5] Ansorge R. Birolo G. James S. A. Telatin A. (2021). Dadaist2: A Toolkit to Automate and Simplify Statistical Analysis and Plotting of Metabarcoding Experiments. *Int. J. Mol. Sci.* 22:5309. 10.3390/IJMS22105309 34069990 PMC8157834

[B6] Asadzadeh M. Ahmad S. Al-Sweih N. Khan Z. (2018). Rapid and Accurate Identification of Candida Albicans and Candida Dubliniensis by Real-Time PCR and Melting Curve Analysis. *Med. Princ. Pract.* 27 543–548. 10.1159/000493426 30176672 PMC6422113

[B7] Auchtung T. A. Fofanova T. Y. Stewart C. J. Nash A. K. Wong M. C. Gesell J. R. (2018). Investigating Colonization of the Healthy Adult Gastrointestinal Tract by Fungi. *MSphere* 3:e92–e18. 10.1128/mSphere.00092-18 29600282 PMC5874442

[B8] Bendová B. Piálek J. Ďureje L’ Schmiedová L. Čížková D. Martin J. F. (2020). How Being Synanthropic Affects the Gut Bacteriome and Mycobiome: Comparison of Two Mouse Species with Contrasting Ecologies. *BMC Microbiol.* 20:194. 10.1186/s12866-020-01859-8 32631223 PMC7336484

[B9] Boehme M. Guzzetta K. E. Bastiaanssen T. F. S. Bastiaanssen Gerard M. M. Andreu Gual-Grau S. S. (2021). Microbiota from Young Mice Counteracts Selective Age-Associated Behavioral Deficits. *Nat. Aging* 1 666–676. 10.1038/s43587-021-00093-9 37117767

[B10] Boehme M. van de Wouw M. Bastiaanssen T. F. S. Olavarría-Ramírez L. Lyons K. Fouhy F. (2019). Mid-Life Microbiota Crises: Middle Age Is Associated with Pervasive Neuroimmune Alterations That Are Reversed by Targeting the Gut Microbiome. *Mol. Psychiatry* 25 2567–2583. 10.1038/s41380-019-0425-1 31092898

[B11] Bougnoux M. E. Diogo D. François N. Sendid B. Veirmeire S. Colombel J. F. (2006). Multilocus Sequence Typing Reveals Intrafamilial Transmission and Microevolutions of Candida Albicans Isolates from the Human Digestive Tract. *J. Clin. Microbiol.* 44 1810–1820. 10.1128/JCM.44.5.1810-1820.2006 16672411 PMC1479199

[B12] Boulangé C. L. Neves A. L. Chilloux J. Nicholson J. K. Dumas M. E. (2016). Impact of the Gut Microbiota on Inflammation, Obesity, and Metabolic Disease. *Genome Med.* 8:42. 10.1186/s13073-016-0303-2 27098727 PMC4839080

[B13] Brown G. D. Denning D. W. Gow N. A. Levitz S. M. Netea M. G. White T. C. (2012). Hidden Killers: Human Fungal Infections. *Sci. Transl. Med.* 4:165rv13. 10.1126/SCITRANSLMED.3004404 23253612

[B14] Callahan B. J. McMurdie P. J. Rosen M. J. Han A. W. Johnson A. J. Holmes S. P. (2016). DADA2: High-Resolution Sample Inference from Illumina Amplicon Data. *Nat. Methods* 13 581–583. 10.1038/NMETH.3869 27214047 PMC4927377

[B15] Chen S. Zhou Y. Chen Y. Gu J. (2018). Fastp: An Ultra-Fast All-in-One FASTQ Preprocessor. *Bioinformatics* 34:i884–i890. 10.1093/BIOINFORMATICS/BTY560 30423086 PMC6129281

[B16] Claesson M. J. Cusack S. O’Sullivan O. Greene-Diniz R. de Weerd H. Flannery E. (2011). Composition, Variability, and Temporal Stability of the Intestinal Microbiota of the Elderly. *Proc.Nat. Acad. Sci. U.S.A.* 108 4586–4591. 10.1073/pnas.1000097107 20571116 PMC3063589

[B17] Clark R. I. Salazar A. Yamada R. Fitz-Gibbon S. Morselli M. Alcaraz J. (2015). Distinct Shifts in Microbiota Composition during Drosophila Aging Impair Intestinal Function and Drive Mortality. *Cell Rep.* 12 1656–1667. 10.1016/j.celrep.2015.08.004 26321641 PMC4565751

[B18] Conti H. R. Huppler A. R. Whibley N. Gaffen S. L. (2014). Animal Models for Candidiasis. *Curr. Protoco. Immunol.* 105 19.6.1-19.6.17. 10.1002/0471142735.IM1906S105 24700323 PMC4088949

[B19] Dalle F. Wächtler B. L’Ollivier C. Holland G. Bannert N. Wilson D. (2010). Cellular Interactions of Candida Albicans with Human Oral Epithelial Cells and Enterocytes. *Cell. Microbiol* 12 248–271. 10.1111/J.1462-5822.2009.01394.X 19863559

[B20] Dhariwal A. Chong J. Habib S. King I. L. Agellon L. B. Xia J. (2017). MicrobiomeAnalyst: A Web-Based Tool for Comprehensive Statistical, Visual and Meta-Analysis of Microbiome Data. *Nucleic Acids Res.* 45:W180–W188. 10.1093/NAR/GKX295 28449106 PMC5570177

[B21] Dominy S. S. Lynch C. Ermini F. Benedyk M. Marczyk A. Konradi A. (2019). *Porphyromonas gingivalis* in Alzheimer’s disease brains: evidence for disease causation and treatment with small-molecule inhibitors. *Sci. Adv.* 5:eaau3333. 10.1126/sciadv.aau3333 30746447 PMC6357742

[B22] Doron I. Leonardi I. Iliev I. D. (2019). Profound Mycobiome Differences between Segregated Mouse Colonies Do Not Influence Th17 Responses to a Newly Introduced Gut Fungal Commensal. *Fungal Genet. Biol.?* 127:45. 10.1016/J.FGB.2019.03.001 30849443 PMC6659114

[B23] Drummond R. A. Desai J. V. Ricotta E. E. Swamydas M. Deming C. Conlan S. (2022). Long-Term Antibiotic Exposure Promotes Mortality after Systemic Fungal Infection by Driving Lymphocyte Dysfunction and Systemic Escape of Commensal Bacteria. *Cell Host Microbe.* [Epub ahead of print]. 10.1016/J.CHOM.2022.04.013 35568028 PMC9283303

[B24] Erwig L. P. Gow N. A. (2016). Interactions of Fungal Pathogens with Phagocytes. *Nat. Rev. Microbiol.* 14 163–176. 10.1038/NRMICRO.2015.21 26853116

[B25] Field L. H. Pope L. M. Cole G. T. Guentzel M. N. Berry L. J. (1981). Persistence and Spread of Candida Albicans after Intragastric Inoculation of Infant Mice. *Infect. Immun.* 31 783–791. 10.1128/IAI.31.2.783-791.1981 7012021 PMC351378

[B26] Forbes J. D. Bernstein C. N. Tremlett H. Van Domselaar G. Knox N. C. (2018). A Fungal World: Could the Gut Mycobiome Be Involved in Neurological Disease? *Front. Microbiol.* 9:3249. 10.3389/FMICB.2018.03249 30687254 PMC6333682

[B27] Fransen F. van Beek A. A. Borghuis T. Aidy S. E. Hugenholtz F. van der Gaast-de Jongh C. (2017). Aged Gut Microbiota Contributes to Systemical Inflammaging after Transfer to Germ-Free Mice. *Front. Immunol.* 8:1385. 10.3389/fimmu.2017.01385 29163474 PMC5674680

[B28] Fung T. C. Olson C. A. Hsiao E. Y. (2017). Interactions between the Microbiota, Immune and Nervous Systems in Health and Disease. *Nat. Neurosci.* 20 145–155. 10.1038/nn.4476 28092661 PMC6960010

[B29] Gardes M. Bruns T. D. (1993). ITS Primers with Enhanced Specificity for Basidiomycetes–Application to the Identification of Mycorrhizae and Rusts. *Mol. Ecol.* 2 113–118. 10.1111/J.1365-294X.1993.TB00005.X 8180733

[B30] Gottfredsson M. Perfect J. R. (2000). Fungal Meningitis. *Semin. Neurol.* 20 307–322. 10.1055/S-2000-9394/ID/40 11051295

[B31] Gouba N. Drancourt M. (2015). Digestive Tract Mycobiota: A Source of Infection. *Med. Mal. Infect.* 45 9–16. 10.1016/J.MEDMAL.2015.01.007 25684583

[B32] Gow N. A. van de Veerdonk F. L. Brown A. J. Netea M. G. (2011). Candida Albicans Morphogenesis and Host Defence: Discriminating Invasion from Colonization. *Nat. Rev. Microbiol.* 10 112–122. 10.1038/nrmicro2711 22158429 PMC3624162

[B33] Hallen-Adams H. E. Kachman S. D. Kim J. Legge R. M. Martínez I. (2015). Fungi Inhabiting the Healthy Human Gastrointestinal Tract: A Diverse and Dynamic Community. *Fungal Ecol.* 15 9–17. 10.1016/J.FUNECO.2015.01.006

[B34] Hammond C. J. Hallock L. R. Howanski R. J. Appelt D. M. Little C. S. Balin B. J. (2010). Immunohistological Detection of Chlamydia Pneumoniae in the Alzheimer’s Disease Brain. *BMC Neurosci.* 11:121. 10.1186/1471-2202-11-121 20863379 PMC2949767

[B35] Huang W. S. Yang T. Y. Shen W. C. Lin C. L. Lin M. C. Kao C. H. (2014). Association between *Helicobacter Pylori* Infection and Dementia. *J. Clin. Neurosci.* 21 1355–1358. 10.1016/J.JOCN.2013.11.018 24629396

[B36] Huseyin C. E. Rubio R. C. O’Sullivan O. Cotter P. D. Scanlan P. D. (2017). The Fungal Frontier: A Comparative Analysis of Methods Used in the Study of the Human Gut Mycobiome. *Front. Microbiol.* 8:1432. 10.3389/fmicb.2017.01432 28824566 PMC5534473

[B37] Jakobsson H. E. Rodríguez-Piñeiro A. M. Schütte A. Ermund A. Boysen P. Bemark M. (2015). The Composition of the Gut Microbiota Shapes the Colon Mucus Barrier. *EMBO Rep.* 16 164–177. 10.15252/EMBR.201439263 25525071 PMC4328744

[B38] James S. A. Phillips S. Telatin A. Baker D. Ansorge R. Clarke P. (2020). Preterm Infants Harbour a Rapidly Changing Mycobiota That Includes Candida Pathobionts. *J. Fungi* 6:273. 10.3390/JOF6040273 33182444 PMC7712117

[B39] Jayasudha R. Das T. Kalyana Chakravarthy S. Sai Prashanthi G. Bhargava A. Tyagi M. (2020). Gut Mycobiomes Are Altered in People with Type 2 Diabetes Mellitus and Diabetic Retinopathy. *PLoS One* 15:e0243077. 10.1371/JOURNAL.PONE.0243077 33259537 PMC7707496

[B40] Johansson M. E. Gustafsson J. K. Holmén-Larsson J. Jabbar K. S. Xia L. Xu H. (2014). Bacteria Penetrate the Normally Impenetrable Inner Colon Mucus Layer in Both Murine Colitis Models and Patients with Ulcerative Colitis. *Gut* 63 281–291. 10.1136/GUTJNL-2012-303207/-/DC1 23426893 PMC3740207

[B41] Kavanaugh N. L. Zhang A. Q. Nobile C. J. Johnson A. D. Ribbeck K. (2014). Mucins Suppress Virulence Traits of Candida Albicans. *MBio* 5 1911–1925. 10.1128/MBIO.01911-14/SUPPL_FILE/MBO005142043S1.DOCX PMC4235211 25389175

[B42] Kinneberg K. M. Bendel C. M. Jechorek R. P. Cebelinski E. A. Gale C. A. Berman J. G. (1999). Effect of INT1 Gene on Candida Albicans Murine Intestinal Colonization. *J. Surgical Res.* 87 245–251. 10.1006/JSRE.1999.5755 10600356

[B43] Koh A. Y. (2013). Murine Models of Candida Gastrointestinal Colonization and Dissemination. *Eukaryot. Cell* 12 1416–1422. 10.1128/EC.00196-13 24036344 PMC3837944

[B44] Koh A. Y. Köhler J. R. Coggshall K. T. Van Rooijen N. Pier G. B. (2008). Mucosal Damage and Neutropenia Are Required for Candida Albicans Dissemination. *PLoS Pathogens* 4:e35. 10.1371/journal.ppat.0040035 18282097 PMC2242836

[B45] Kurtzman C. P. Robnett C. J. Ward J. M. Brayton C. Gorelick P. Walsh T. J. (2005). Multigene Phylogenetic Analysis of Pathogenic Candida Species in the Kazachstania (Arxiozyma) Telluris Complex and Description of Their Ascosporic States as Kazachstania Bovina Sp. Nov., K. Heterogenica Sp. Nov., K. Pintolopesii Sp. Nov., and K. Slooffiae Sp. Nov. *J. Clin. Microbiol.* 43 101–111. 10.1128/JCM.43.1.101-111.2005 15634957 PMC540161

[B46] Lagkouvardos I. Fischer S. Kumar N. Clavel T. (2017). Rhea: A Transparent and Modular R Pipeline for Microbial Profiling Based on 16S RRNA Gene Amplicons. *PeerJ* 5:e2836. 10.7717/PEERJ.2836 28097056 PMC5234437

[B47] Langille M. G. Meehan C. J. Koenig J. E. Dhanani A. S. Rose R. A. Howlett S. E. (2014). Microbial Shifts in the Aging Mouse Gut. *Microbiome* 2:50. 10.1186/s40168-014-0050-9 25520805 PMC4269096

[B48] Li J. Jia H. Cai X. Zhong H. Feng Q. Sunagawa S. (2014). An Integrated Catalog of Reference Genes in the Human Gut Microbiome. *Nat. Biotechnol.* 32 834–841. 10.1038/NBT.2942 24997786

[B50] Ling Z. Zhu M. Liu X. Shao L. Cheng Y. Yan X. (2020). Fecal Fungal Dysbiosis in Chinese Patients With Alzheimer’s Disease. *Front. Cell Dev. Biol.* 8:631460. 10.3389/FCELL.2020.631460 33585471 PMC7876328

[B51] Lionakis M. S. Lim J. K. Lee C. C. Murphy P. M. (2011). Organ-Specific Innate Immune Responses in a Mouse Model of Invasive Candidiasis. *J. Innate Immunity* 3:180. 10.1159/000321157 21063074 PMC3072204

[B52] Maier L. Goemans C. V. Wirbel J. Kuhn M. Eberl C. Pruteanu M. (2021). Unravelling the collateral damage of antibiotics on gut bacteria. *Nature* 599, 120–124. 10.1038/s41586-021-03986-2 34646011 PMC7612847

[B53] Maier L. Pruteanu M. Kuhn M. Zeller G. Telzerow A. Anderson E. E. (2018). Extensive Impact of Non-Antibiotic Drugs on Human Gut Bacteria. *Nature* 555 623–628. 10.1038/nature25979 29555994 PMC6108420

[B92] Mawanda F. Wallace R. (2013). Can infections cause Alzheimer’s disease? *Epidemiol Rev.* 35, 161–180. 10.1093/epirev/mxs007 23349428 PMC4707877

[B54] Mellado E. Cuenca-Estrella M. Regadera J. González M. Díaz-Guerra T. M. Rodríguez-Tudela J. L. (2000). Sustained Gastrointestinal Colonization and Systemic Dissemination by Candida Albicans, Candida Tropicalis and Candida Parapsilosis in Adult Mice. *Diagn. Microbiol. Infect. Dis.* 38 21–28. 10.1016/S0732-8893(00)00165-6 11025180

[B55] Mims T. S. Abdallah Q. A. Stewart J. D. Watts S. P. White C. T. Rousselle T. V. (2021). The Gut Mycobiome of Healthy Mice Is Shaped by the Environment and Correlates with Metabolic Outcomes in Response to Diet. *Commun. Biol.* 4:281. 10.1038/s42003-021-01820-z 33674757 PMC7935979

[B56] Nagpal R. Neth B. J. Wang S. Mishra S. P. Craft S. Yadav H. (2020). Gut Mycobiome and Its Interaction with Diet, Gut Bacteria and Alzheimer’s Disease Markers in Subjects with Mild Cognitive Impairment: A Pilot Study. *EBioMedicine* 59:102950. 10.1016/j.ebiom.2020.102950 32861197 PMC7475073

[B57] Nash A. K. Stewart C. J. Smith D. P. Muzny D. M. Metcalf G. A. Gesell J. R. (2017). The Gut Mycobiome of the Human Microbiome Project Healthy Cohort. *Microbiome* 5:53. 10.1186/s40168-017-0373-4 29178920 PMC5702186

[B58] Nilsson R. H. Larsson K. H. Taylor A. F. S. Bengtsson-Palme J. Jeppesen T. S. Schigel D. (2019). The UNITE Database for Molecular Identification of Fungi: Handling Dark Taxa and Parallel Taxonomic Classifications. *Nucleic Acids Res.* 47:D259–D264. 10.1093/NAR/GKY1022 30371820 PMC6324048

[B59] Noble S. M. Gianetti B. A. Witchley J. N. (2016). Candida Albicans Cell-Type Switching and Functional Plasticity in the Mammalian Host. *Nat. Rev. Microbiol.* 15 96–108. 10.1038/NRMICRO.2016.157 27867199 PMC5957277

[B60] O’Toole P. W. Jeffery I. B. (2015). Gut Microbiota and Aging. *Science* 350 1214–1215. 10.1126/science.aac8469 26785481

[B61] Pande K. Chen C. Noble S. M. (2013). Passage through the Mammalian Gut Triggers a Phenotypic Switch That Promotes Candida Albicans Commensalism. *Nat. Genet.* 45:1088. 10.1038/NG.2710 23892606 PMC3758371

[B62] Parker A. Boloko L. Moolla M. S. Ebrahim N. Ayele B. T. Broadhurst A. G. B. (2021). Heterochronic fecal microbiota transfer reverses hallmarks of the aging murine gut, eye and brain. *SSRN Electron. J.* 10.2139/SSRN.3811833

[B63] Parker A. Boloko L. Moolla M. S. Ebrahim N. Ayele B. T. Broadhurst A. G. B. (2022). Fecal Microbiota Transfer between Young and Aged Mice Reverses Hallmarks of the Aging Gut, Eye, and Brain. *Microbiome* 10:68. 10.1186/S40168-022-01243-W 35501923 PMC9063061

[B64] Pfaller M. A. Diekema D. J. (2007). Epidemiology of Invasive Candidiasis: A Persistent Public Health Problem. *Clin. Microbiol. Rev.* 20 133–163. 10.1128/CMR.00029-06 17223626 PMC1797637

[B65] Pisa D. Alonso R. Juarranz A. Rábano A. Carrasco L. (2015a). Direct Visualization of Fungal Infection in Brains from Patients with Alzheimer’s Disease. *J. Alzheimer’s Dis.* 43 613–624. 10.3233/JAD-141386 25125470

[B66] Pisa D. Alonso R. Rábano A. Rodal I. Carrasco L. (2015b). Different Brain Regions Are Infected with Fungi in Alzheimer’s Disease. *Sci. Rep.* 5:15015. 10.1038/srep15015 26468932 PMC4606562

[B67] Price M. N. Dehal P. S. Arkin A. P. (2009). FastTree: Computing Large Minimum Evolution Trees with Profiles Instead of a Distance Matrix. *Mol. Biol. Evol.* 26 1641–1650. 10.1093/MOLBEV/MSP077 19377059 PMC2693737

[B68] Prieto D. Pla J. (2015). Distinct Stages during Colonization of the Mouse Gastrointestinal Tract by Candida Albicans. *Front. Microbiol.* 6:792. 10.3389/FMICB.2015.00792 26300861 PMC4525673

[B69] Qin J. Li R. Raes J. Arumugam M. Burgdorf K. S. Manichanh C. (2010). A Human Gut Microbial Gene Catalogue Established by Metagenomic Sequencing. *Nature* 464 59–65. 10.1038/NATURE08821 20203603 PMC3779803

[B70] Raimondi S. Amaretti A. Gozzoli C. Simone M. Righini L. Candeliere F. (2019). Longitudinal Survey of Fungi in the Human Gut: ITS Profiling, Phenotyping, and Colonization. *Front. Microbiol.* 10:1575. 10.3389/FMICB.2019.01575/BIBTEX PMC6636193 31354669

[B71] Richard M. L. Sokol H. (2019). The Gut Mycobiota: Insights into Analysis, Environmental Interactions and Role in Gastrointestinal Diseases. *Nat. Rev. Gastroenterol. Hepatol.* 16 331–345. 10.1038/s41575-019-0121-2 30824884

[B72] Saji N. Murotani K. Hisada T. Tsuduki T. Sugimoto T. Kimura A. (2019). The Relationship between the Gut Microbiome and Mild Cognitive Impairment in Patients without Dementia: A Cross-Sectional Study Conducted in Japan. *Sci. Rep.* 9:19227. 10.1038/S41598-019-55851-Y 31852995 PMC6920432

[B73] Schindelin J. Arganda-Carreras I. Frise E. Kaynig V. Longair M. Pietzsch T. (2012). Fiji: An Open-Source Platform for Biological-Image Analysis. *Nat. Methods* 9 676–682. 10.1038/nmeth.2019 22743772 PMC3855844

[B74] Schofield D. A. Westwater C. Balish E. (2005). Short Communication Correspondence Divergent Chemokine, Cytokine and â-Defensin Responses to Gastric Candidiasis in Immunocompetent C57BL/6 and BALB/c Mice. *J. Med. Microbiol.* 54 87–92. 10.1099/jmm.0.45755-0 15591261

[B75] Scott K. A. Ida M. Peterson V. L. Prenderville J. A. Moloney G. M. Izumo T. (2017). Revisiting Metchnikoff: Age-Related Alterations in Microbiota-Gut-Brain Axis in the Mouse. *Brain Behav. Immunity* 65 20–32. 10.1016/j.bbi.2017.02.004 28179108

[B76] Segal E. Frenkel M. (2018). Experimental In Vivo Models of Candidiasis. *J. Fungi* 4:21. 10.3390/JOF4010021 29415485 PMC5872324

[B77] Sender R. Fuchs S. Milo R. (2016). Revised Estimates for the Number of Human and Bacteria Cells in the Body. *PLoS Biol.* 14:21. 10.1371/JOURNAL.PBIO.1002533 27541692 PMC4991899

[B78] Sievers F. Higgins D. G. (2021). The Clustal Omega Multiple Alignment Package. *Methods Mol. Biol.* 2231 3–16. 10.1007/978-1-0716-1036-7_1 33289883

[B79] Snippert H. J. Schepers A. G. Delconte G. Siersema P. D. Clevers H. (2011). Slide Preparation for Single-Cell–Resolution Imaging of Fluorescent Proteins in Their Three-Dimensional near-Native Environment. *Nat. Protoc.* 6 1221–1228. 10.1038/nprot.2011.365 21799490

[B80] Strati F. Di Paola M. Stefanini I. Albanese D. Rizzetto L. Lionetti P. (2016). Age and Gender Affect the Composition of Fungal Population of the Human Gastrointestinal Tract. *Front. Microbiol.* 7:1227. 10.3389/FMICB.2016.01227/BIBTEX PMC4971113 27536299

[B81] Telatin A. Fariselli P. Birolo G. (2021). SeqFu: A Suite of Utilities for the Robust and Reproducible Manipulation of Sequence Files. *Bioengineering* 8:59. 10.3390/bioengineering8050059 34066939 PMC8148589

[B82] Tetz G. Pinho M. Pritzkow S. Mendez N. Soto C. Tetz V. (2020). Bacterial DNA promotes tau aggregation. *Sci. Rep.* 10:2369. 10.1038/s41598-020-59364-x 32047247 PMC7012890

[B83] Thevaranjan N. Puchta A. Schulz C. Naidoo A. Szamosi J. C. Verschoor C. P. (2017). Age-Associated Microbial Dysbiosis Promotes Intestinal Permeability, Systemic Inflammation, and Macrophage Dysfunction. *Cell Host Microbe* 21:455–466.e4. 10.1016/j.chom.2017.03.002 28407483 PMC5392495

[B84] Vich Vila A. Collij V. Sanna S. Sinha T. Imhann F. Bourgonje A. R. (2020). Impact of Commonly Used Drugs on the Composition and Metabolic Function of the Gut Microbiota. *Nat. Commun.* 11:362. 10.1038/s41467-019-14177-z 31953381 PMC6969170

[B85] Vogt N. M. Kerby R. L. Dill-McFarland K. A. Harding S. J. Merluzzi A. P. Johnson S. C. (2017). Gut Microbiome Alterations in Alzheimer’s Disease. *Sci. Rep.* 7:13537. 10.1038/S41598-017-13601-Y 29051531 PMC5648830

[B86] Ward T. L. Dominguez-Bello M. G. Heisel T. Al-Ghalith G. Knights D. Gale C. A. (2018). Development of the Human Mycobiome over the First Month of Life and across Body Sites. *MSystems* 3:e00140-17. 10.1128/MSYSTEMS.00140-17 29546248 PMC5840654

[B87] White T. J. Bruns T. Lee S. Taylor J. (1990). “Amplification And Direct Sequencing Of Fungal Ribosomal Rna Genes For Phylogenetics,” in *PCR Protocols: a Guide to Methods and Applications*, eds Innis M. A. Gelfand D. H. Sninsky J. J. White T. J. (New York, N.Y: Academic Press, Inc), 315–322.

[B88] Wiesner S. M. Jechorek R. P. Garni R. M. Bendel C. M. Wells C. L. (2001). Gastrointestinal Colonization by Candida Albicans Mutant Strains in Antibiotic-Treated Mice. *Clin. Diagnostic Lab. Immunol.* 8 192–195. 10.1128/CDLI.8.1.192-195.2001 11139219 PMC96034

[B89] Wu Y. Du S. Johnson J. L. Tung H. Y. Landers C. T. Liu Y. (2019). Microglia and Amyloid Precursor Protein Coordinate Control of Transient Candida Cerebritis with Memory Deficits. *Nat. Commun.* 10:58. 10.1038/s41467-018-07991-4 30610193 PMC6320369

[B90] Yatsunenko T. Rey F. E. Manary M. J. Trehan I. Dominguez-Bello M. G. Contreras M. (2012). Human Gut Microbiome Viewed across Age and Geography. *Nature* 486 222–227. 10.1038/nature11053 22699611 PMC3376388

[B91] Zhuang Z. Q. Shen L. L. Li W. W. Fu X. Zeng F. Gui L. (2018). Gut Microbiota Is Altered in Patients with Alzheimer’s Disease. *J. Alzheimer’s Dis.?* 63 1337–1346. 10.3233/JAD-180176 29758946

