# Supplementary figures and images for: Absence of Bacteria Permits Fungal Gut-To-Brain Translocation and Invasion in Germfree Mice but Ageing Alone Does Not Drive Pathobiont Expansion in Conventionally Raised Mice
Source: Front Aging Neurosci. 2022 Jul 18;14:828429. doi: 10.3389/fnagi.2022.828429 (PMC9339909; doi:10.3389/fnagi.2022.828429)

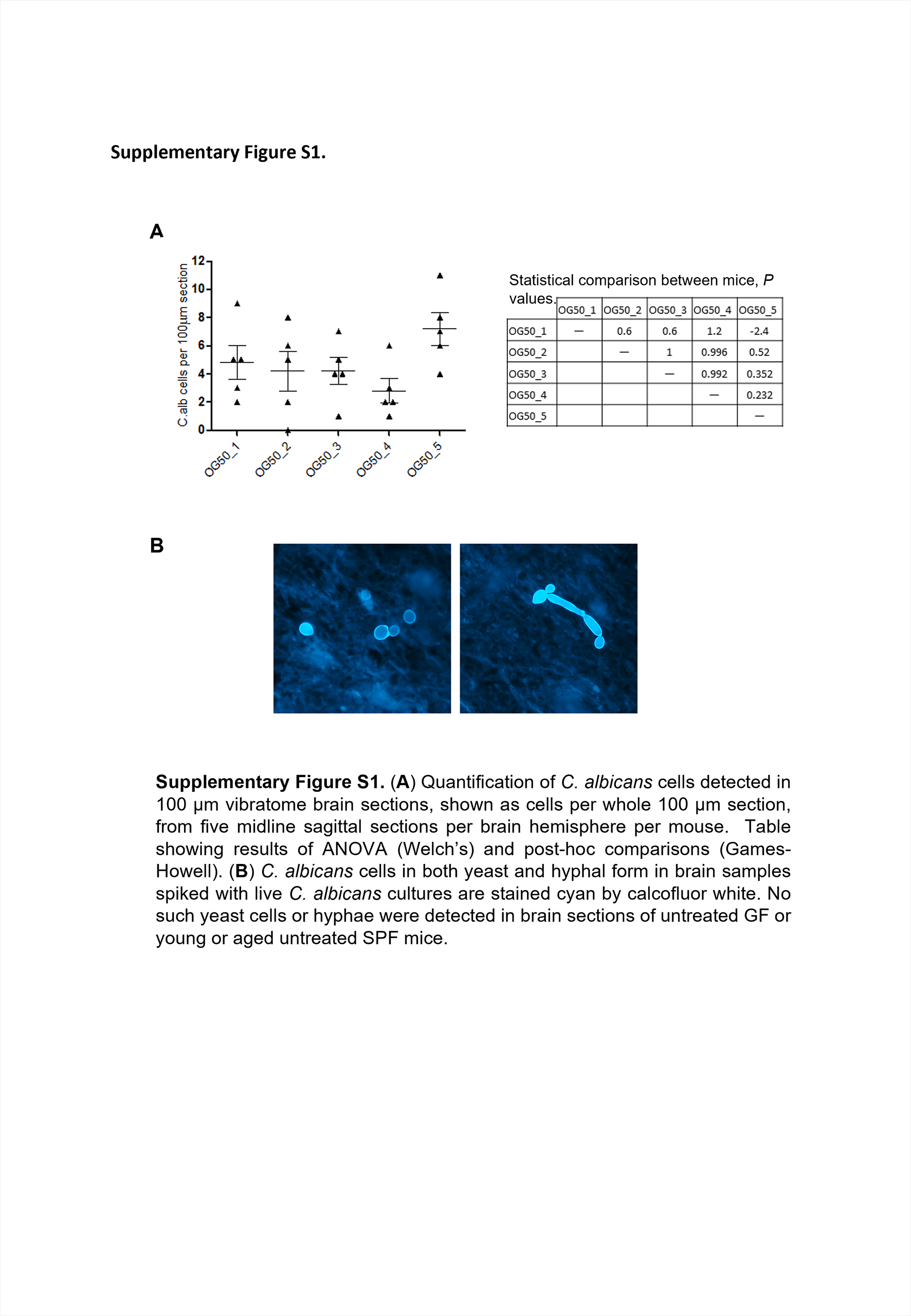

Supplement: Supplementary file 1 [file Image_1.tif]

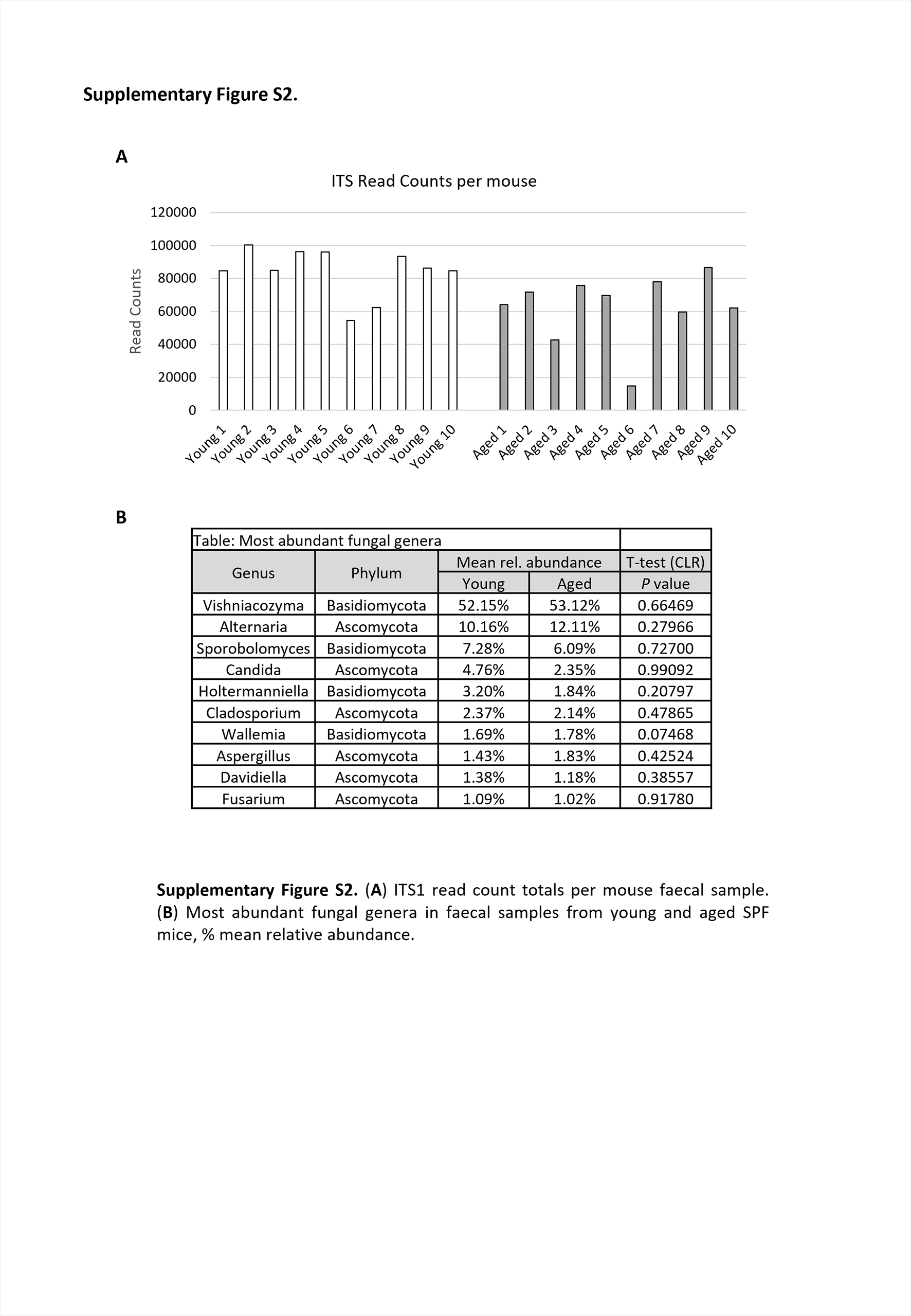

Supplement: Supplementary file 2 [file Image_2.tif]

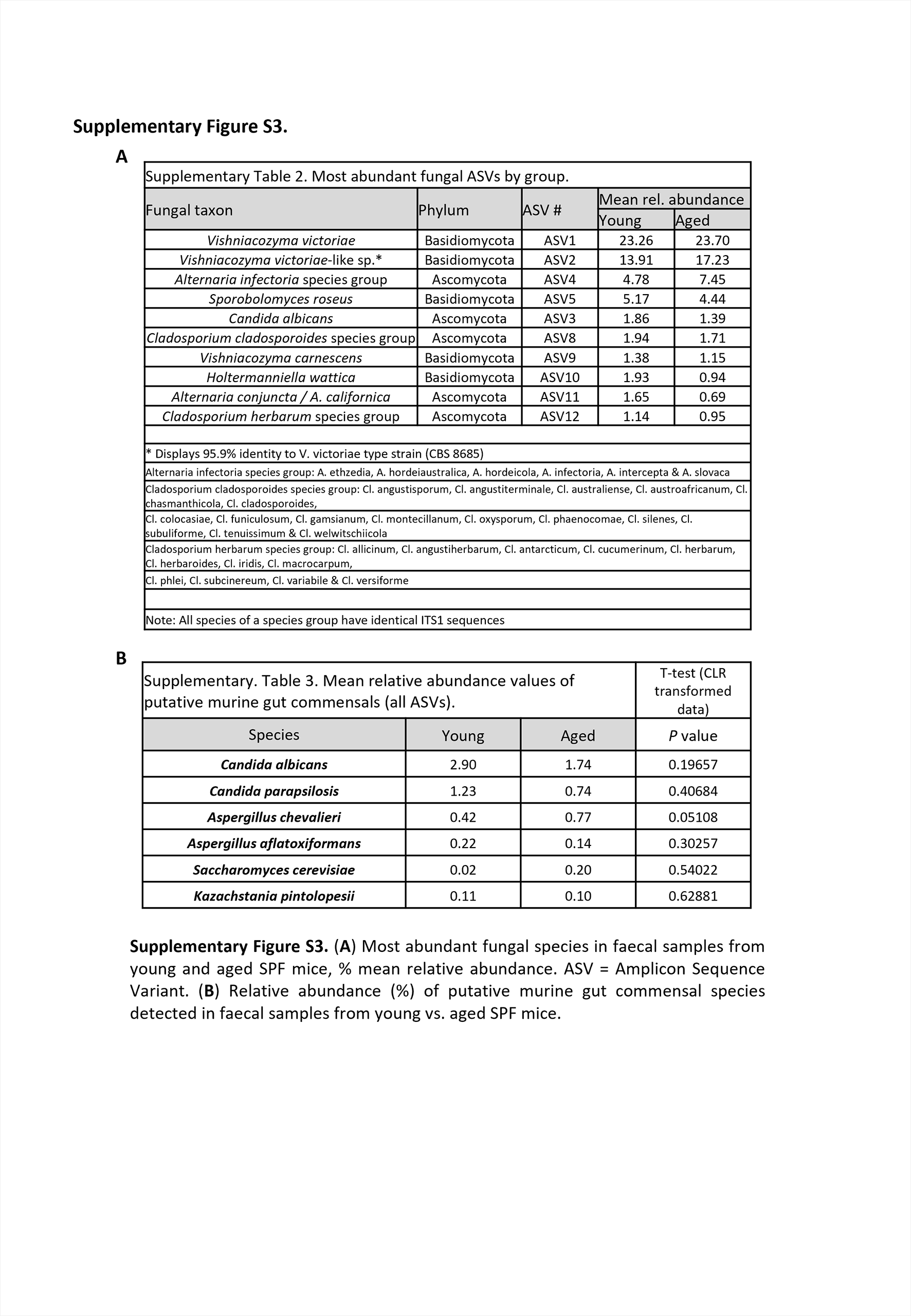

Supplement: Supplementary file 3 [file Image_3.tif]

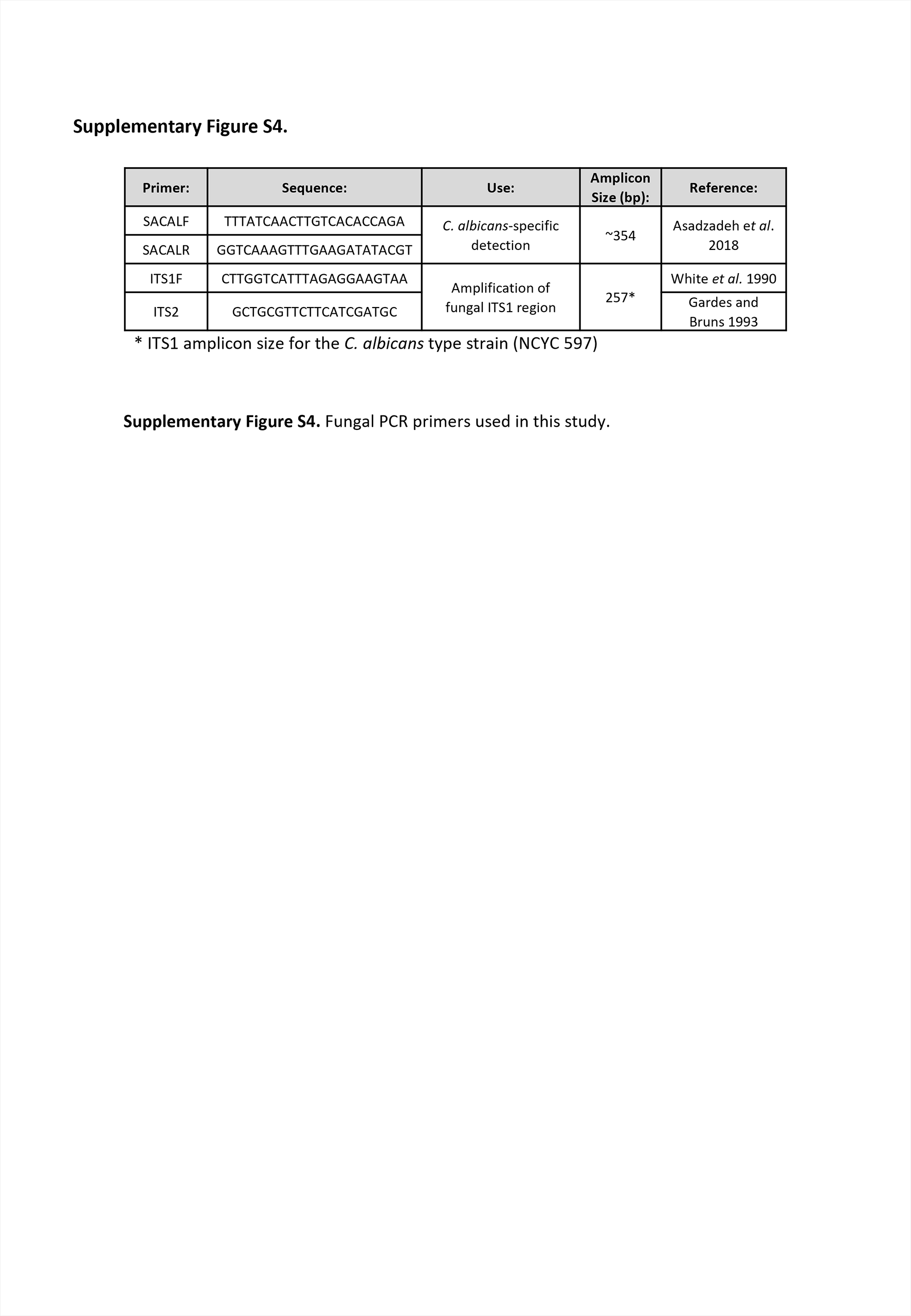

Supplement: Supplementary file 4 [file Image_4.tif]
